# A multi-year experiment shows that lower precipitation predictability encourages plants’ early life stages and enhances population viability

**DOI:** 10.7717/peerj.6443

**Published:** 2019-03-08

**Authors:** Martí March-Salas, Patrick S. Fitze

**Affiliations:** 1 Department of Biodiversity and Evolutionary Biology, Museo Nacional de Ciencias Naturales (MNCN-CSIC), Madrid, Spain; 2 Department of Biodiversity and Ecologic Restoration, Instituto Pirenaico de Ecología (IPE-CSIC), Jaca, Spain; 3 Escuela Internacional de Doctorado, Universidad Rey Juan Carlos (URJC), Madrid, Spain

**Keywords:** Climatic variability, Environmental predictability, Life-cycle, Vital rates, Transgenerational response, Survival, *Onobrychis viciifolia*, *Papaver rhoeas*, Population growth rate, Seedling emergence

## Abstract

Climate change is a key factor that may cause the extinction of species. The associated reduced weather predictability may alter the survival of plants, especially during their early life stages, when individuals are most fragile. While it is expected that extreme weather events will be highly detrimental for species, the effects of more subtle environmental changes have been little considered. In a four-year experiment on two herbaceous plants, *Papaver rhoeas* and *Onobrychis viciifolia*, we manipulated the predictability of precipitation by changing the temporal correlation of precipitation events while maintaining average precipitation constant, leading to more and less predictable treatments. We assessed the effect of predictability on plant viability in terms of seedling emergence, survival, seed production, and population growth rate. We found greater seedling emergence, survival, and population growth for plants experiencing lower intra-seasonal predictability, but more so during early compared to late life stages. Since predictability levels were maintained across four generations, we have also tested whether descendants exhibited transgenerational responses to previous predictability conditions. In *P. rhoeas*, descendants had increased the seedling emergence compared to ancestors under both treatments, but more so under lower precipitation predictability. However, higher predictability in the late treatment induced higher survival in descendants, showing that these conditions may benefit long-term survival. This experiment highlights the ability of some plants to rapidly exploit environmental resources and increase their survival under less predictable conditions, especially during early life stages. Therefore, this study provides relevant evidence of the survival capacity of some species under current and future short-term environmental alterations.

## Introduction

Species survival is closely linked to environmental changes (Thomas et al., 2004), which are increasing due to anthropogenic influences. If these changes exceed a species’ tolerance limits, many individuals will not be able to adapt and will disappear (Gonzalez et al., 2013). This is especially true for plants, since, as sessile organisms, they cannot move away from unfavorable habitats. Rapid adaptive capacity is therefore becoming increasingly important for plants in rapidly altering environments (Loarie et al., 2009). Mean climatic conditions usually change very gradually (Katz & Brown, 1992), which can allow plants to develop coping strategies. Reduced environmental predictability caused by increasing climatic variability, however, might be more difficult to buffer against. While several studies have demonstrated that organisms are particularly sensitive to climatic extremes (Parmesan, Root & Willig, 2000, and references therein), still little is known about the effects of the subtle decreases of environmental predictability driven by climate change (Intergovernmental Panel on Climate Change (IPCC), 2014).

Tolerance to less predictable environmental conditions may differ through the multiple plant life stages (Burghardt et al., 2015). Environmental conditions that plants experience during their early stages (i.e., seedlings) can potentially have strong effects on plant germination, survival, and performance. Plants are more vulnerable at that stage than during adult phase, and small environmental changes can lead to higher mortality rates (Donohue et al., 2010). The effects on developmental stages could trigger in changes in performance during subsequent life stages (Jonsson & Jonsson, 2014), in fitness (Cam, Monnat & Hines, 2003), and even on the success of future generations (Walck et al., 2011; Burton & Metcalfe, 2014). Therefore, it is important to identify when plants are more affected by weather alterations, and if there is a relationship between the differential effect on life stages and the future success of plants. Thus, to understand the response of individuals to lower environmental predictability, Lawson et al. (2015) suggested testing their effects on each life stage component, including birth, death, and development. Germination (i.e., seedling emergence) is the first step of plant life, and water availability is a crucial resource for this process (Classen et al., 2010; Walck et al., 2011). Seeds need sufficient humidity to emerge, and certain constancy in wet conditions could induce greater seedling emergence (Finch-Savage, Steckel & Phelps, 1998). However, emergence of seedlings could also benefit from several oscillations in humidity conditions (Gremer, Kimball & Venable, 2016). If plants could endure inconsistent conditions during early stages, they may be able to survive until the end of their biological cycle (Donohue et al., 2010). In this regard, weather alterations during the early stages of development may have stronger effects on the survival of individuals than during other life stages. However, early life stages are rarely considered in field experiments (Postma & Ågren, 2016). Understanding how early survival is shaped by lower environmental predictability is very relevant for potential species adaptation.

Whereas highly predictable environments are expected to help maintain constant population sizes in the long-term and favors plants adaptation (Olson-Manning, Wagner & Mitchell-Olds, 2012), ecologists commonly assume population decreases in habitats of lower predictability (Stearns, 1992; Lande, Engen & Saether, 2003), since they anticipate a depression in viability and fitness components (Anderson, 2016). However, other studies contradict this assumption, since they found that environmental variability and associated reduced predictability maintains diversity (Adler et al., 2006; Gherardi & Sala, 2015; Jones et al., 2016) and may lead to an increase of phenotypic plasticity (Ghalambor et al., 2007; Merilä & Hendry, 2014; March-Salas et al., 2018, unpublished data). Moreover, if plants of the preceding generation (i.e., ancestors) were growing under weather variations, this may lead their descendants to assimilate these conditions as the most appropriate to develop, or at least, ancestors could prevent descendants of weather alterations (e.g., via maternal effects). Thus, environmental conditions would be less harmful than models and theories project (Chevin, Lande & Mace, 2010; Botero et al., 2015). The effect of environmental predictability on intra- and inter-generations is still largely underappreciated (Reed et al., 2010), and these effects are key to foresee the future consequences of the growing weather instability for individuals, and even populations.

Here, we tested in a four-year experiment with two plant species, *Onobrychis viciifolia* and *Papaver rhoeas*, whether and how changes in precipitation predictability: (1) induced immediate responses in seedling emergence, early and late plant survival, reproductive individual rate (i.e. seed production) and plot-population growth; (2) affected plants in different life stages (early and late stage), and (3) led to changes over time in the number of survivals across multiple-generation (i.e., transgenerational responses). Therefore, testing the hypotheses 1 and 3 will allow us to shed light on the survival of some plant species in the face of an expected lower environmental predictability. Moreover, testing the hypothesis 2 will unravel which life-cycle stage is prone to suffer these weather alterations and their potential impact for future plants viability.

## Materials and methods

### Experimental system and procedures

Seeds of *P. rhoeas* L. (common poppy; Papaveraceae) and *O. viciifolia* Scop. (common sainfoin; Fabaceae) were sown in natural environments located at the experimental field station ‘El Boalar’ (42°33′N, 0°37′W, 705 m.a.s.l.; IPE-CSIC, Jaca, Huesca, Spain) and exposed to different precipitation-predictability regimes (see below) during four consecutive years (2012–2015). *Papaver rhoeas* and *O. viciifolia* were selected as model species because of their similar growth season but different life histories and reproductive strategies (for species details, see ‘Supplementary Material’). Also, wild individuals of both plant species occur on the study site. Seeds of *P. rhoeas* and *O. viciifolia* were obtained in 2011 and were never previously exposed to the experimentally simulated conditions, but still came from geographically close sites with similar climatic conditions. Seeds of *O. viciifolia* originated from a farm located in Castillo de Lerés (23 km apart from the field site) and seeds of *P. rhoeas* from a farm located in the Ebro Valley near Zaragoza (ca. 75 km apart from the field site). Since these seeds were naive with regard to the experimental conditions, they were referred as the ancestral generation (G_0_) and distinguish them from their descendants (G_1–3_; see below).

In 2012, 16 open-air enclosures were established, each with two experimental plots of 1.2 × 6.0 m (Fig. S1). In one of the two plots (per enclosure), we sowed *P. rhoeas* at a depth of one cm, and in the other plot, we sowed *O. viciifolia* at a depth of two cm. The enclosures were surrounded by metal walls and covered by a mesh (mesh width: 1.6 × 1.6 cm) that protected the plants against large herbivores, but which allowed access for insects (including pollinators). Each plot was additionally surrounded with a mosquito mesh (30 cm above ground and 10 cm below ground) to protect against slug predation. Before the beginning of the experiment, we loosened and homogenized the top 30 cm of the soil in each plot. All weeds, roots, and visible seeds were removed to avoid competition with other undesirable plant species, and the ground was smoothed. For each species in different plots, in the first year, randomly selected seeds were sown in 28 positions (i.e., three seeds per position) per plot regularly distributed and separated by 40 cm each (Fig. S1). In subsequent years (2013–2015), when we sowed descendants of the preceding generation, we also always sowed randomly selected seeds of the original ancestral generation in seven of the 28 positions (i.e., four seeds per position) in each plot in order to quantify potential transgenerational responses (i.e., acting as control individuals; see below). Before sowing, to assure that the randomly selected subsamples each year were representative for the entire ancestral seed lot, we statistically tested that there were no significant differences in averages and variances of seed mass of seeds selected or not selected to be used in this experiment, among seeds used in different years, plots, and experimental treatments (*P* ≥ 0.1 in all cases). When the first emerged seedlings reached five cm in *P. rhoeas* and 10 cm in *O. viciifolia*, seedling height and the seedlings maximal diameter were measured to the nearest millimeter. In the case that more than one seedling was present in a given position, one seedling was randomly selected and the other ones were thinned, to avoid competition among seedlings. To this aim, from seedling emergence, we statistically tested by Linear Mixed-effect Models (LMM) that there were no significant differences in days to seedling emergence, seedling height, seedling diameter, and relative growth rate (in height and diameter) among thinned and non-thinned seedlings, and all interactions between thinning and enclosure or thinning treatments were not significant (*P* ≥ 0.8 in all cases).

### Precipitation-predictability treatment

**Figure 1:**
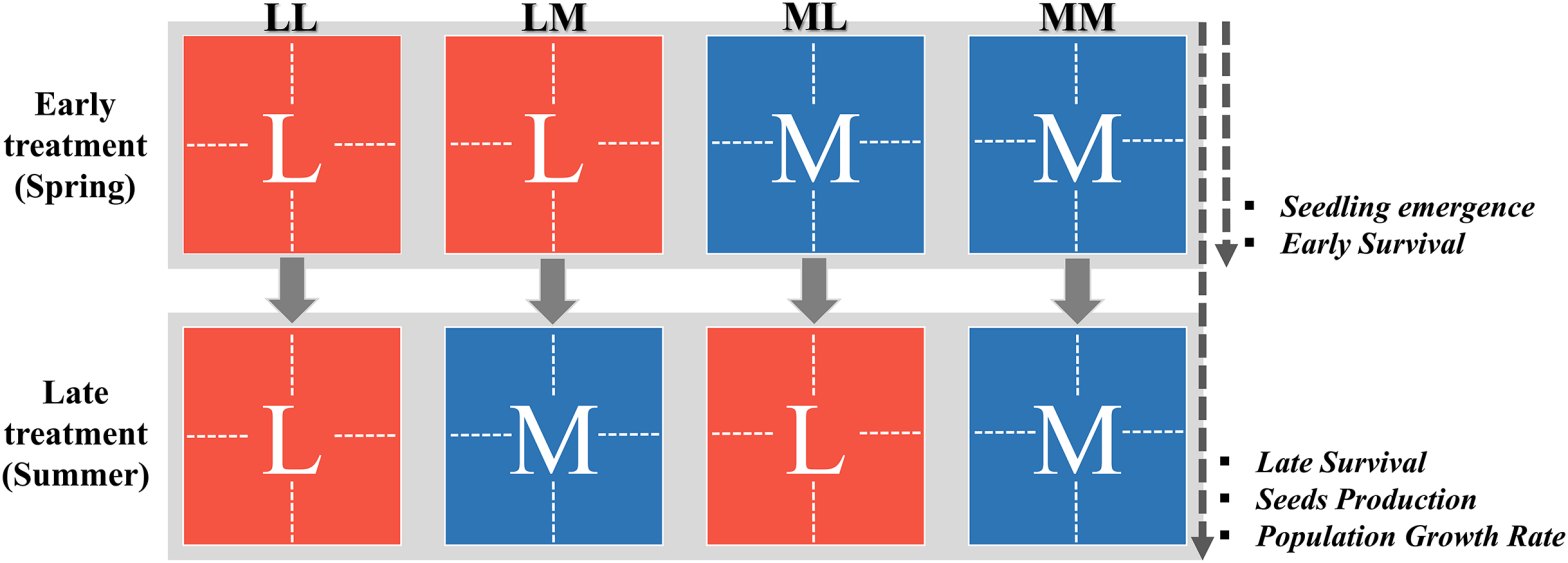
Two-factorial experimental design of the precipitation-predictability treatment. The factors were the early and the late treatment. The ‘Early treatment’ covers the spring period and consisted of two levels: less (L; red color) and more predictable precipitation (M; blue color), each applied to eight enclosures (represented by dotted lines inside the squares). At the end of this treatment (before the switch point between early and late stages; see ‘Materials and Methods’), the seedling emergence and the survival during early stage (i.e., early survival) was measured, and thus, these traits could be affected by the early treatment (see arrow on the right). The ‘Late treatment’ covers the summer period and also consisted of less and more predictable precipitation (i.e., levels). This thus resulted in a two-factorial design with four early treatment-by-late treatment combinations (LL, LM, ML, MM). At the end of the late treatment, we measured the survival during the late stage (i.e., late survival), the seed production and the population growth rate, and thus, these traits could be affected by both early and late treatment (see arrow on the right). This experimental design was applied for 4 years (2012–2015).

Precipitation predictability (i.e., the level of temporal autocorrelation of environmental parameters) was manipulated at two different temporal scales: intra- and inter-seasonally. First, for intra-seasonal predictability, we simulated more (M) and less (L) predictable precipitation treatments by manipulating the timing of precipitation within each week, thus varying the daily predictability of precipitation. In M, the probability and timing of rainfall were more predictable (higher autocorrelation among days), while in L both were less predictable. Treatments were applied during two different periods (i.e., seasons) within each year: from early spring to late spring (spring season), and from early to late summer (summer season). Spring season started in March–April and extended until the end of June, and it was associated with early plant stage. Thus, we hereafter referred it to as ‘early treatment’. Summer season started at the end of June-beginning of July and extended until October, and it was associated with late plant stage. Thus, we hereafter referred it to as ‘late treatment’. Each year, the irrigation treatments started a couple of weeks before sowing and ended after plant harvesting. Each enclosure was irrigated individually using an automatic irrigation system with four sprinklers per enclosure, one in each corner, to provide homogenous precipitation in the whole enclosure. Thus, during the early and late treatment, half of the enclosures (eight of 16) were exposed to M and the other half to L (Fig. 1). The M treatment was imposed by irrigating plots twice a day for 10 min at regular intervals between 9 a.m. and 7 p.m. (i.e., 14 times per week), and the L treatment was imposed by also irrigating 14 times for 10 min but at randomly chosen time points during the week (between 9 a.m. and 7 p.m.). All enclosures were exposed to the same natural background precipitation and thus, enclosures of the more predictable treatment received two rain events (experimental and natural events combined) per day on 76.8% of the days and more than two events per day on 23.2% of the days, whereas enclosures of the less predictable treatment received less than two rain events per day on 30.4% of the days, two events per day on 22.6%, and more than two events per day on 47% of the days. Thus, the timing of precipitation differed between treatments, while the number of precipitation events and the total amount of precipitation were identical (e.g., see Fig. S2). The simulated variance in daily precipitation measured over one week was within the natural limits, since the minimum variance was zero in natural precipitation and in both treatment levels, while the maximum variance did not significantly differ from the natural precipitation, neither in M, nor in L.

Second, inter-seasonal predictability (i.e., the level of autocorrelation between spring and summer) was manipulated by exposing eight enclosures during the late plant stage (summer) to either the same or to the other intra-seasonal predictability regime as during the early plant stage (spring; Fig. 1). This thus resulted in a two-factorial design with four early treatment-by-late treatment combinations (Fig. 1): (1) more predictable during early stage and more predictable during late stage (MM), (2) less predictable in both periods (LL), (3) more predictable during early stage and less predictable during late stage (ML), and (4) less predictable during early stage and more predictable during late stage (LM). Thus, plants were exposed to higher inter-seasonal predictability (MM, LL) or lower inter-seasonal (ML, LM) predictability, or in other words, to a higher or lower autocorrelation of precipitation between early (spring) and late plant stage (summer; Fig. 1). For the changing point between early and late stage, we chose the middle of the phenological life-cycle of both species (i.e., when first’s flower buds appeared). This was, depending on the year, at the end of June or beginning of July, proximate with the change of spring-summer season.

### Testing for transgenerational responses

To test for transgenerational responses with respect to precipitation predictability, offspring/descendant seeds produced by a subset ancestral plants (G_0_) were stored over winter, and a randomly chosen subsample of those seeds (774 for *P. rhoeas* and 252 for *O. viciifolia* from seven to eight maternal lines per treatment combination and species) was sown again in the experimental plots in the subsequent year. No significant differences existed between used and non-used mothers (i.e., selection of progenitors to be used for providing seed progeny in the following year) in mean and variance of emergence time (days), maximum height (in mm), maximum diameter (mm), number and mass of produced seeds, beginning of flowering period, and above-ground and root biomass (g) within treatment combinations, mother enclosure, and treatment × mother enclosure combinations (*P* > 0.2 in all cases) and between used and not used descendant in mean and variance of seed mass (*P* ≥ 0.1 in all cases).

For each of these maternal lines, we kept the treatment combinations (i.e., MM, LL, LM, ML) constant across all generations. Thus, we had four generations exposed to the same conditions: the G_0_ ancestral generation, and the G_1_ (in 2013), G_2_ (in 2014) and G_3_ (in 2015) as descendant generations. While descendants were potentially able to exhibit a transgenerational response, by experimental design, ancestors were unable to do a transgenerational response with respect to the experimental conditions. Thus, differences among ancestors planted in different years represent differences due to variation among years, while differences between ancestors and descendants growing in the same plot and year represent transgenerational responses. Moreover, to avoid local adaptation to specific conditions, of a particular plot beyond the experimental precipitation regime, we sowed the descendants in other plot than where the ancestor had been growing.

### Data collection

From each seed sown, seedling emergence was checked daily during the first four weeks of the experiment to determine the seedling emergence. The plant survival during the early stage was assessed weekly from seedling emergence to the end of the early stage. Alive thinned seedlings (see above) in this period were also considered as survivors. For seedlings that were alive and non-thinned during the early stage, we determined the plant survival during the late stage (i.e., from the end of the early stage until the end of their annual cycle). Seeds were collected weekly when they ripen and the seeds from the same individual were counted. These seeds were included in the same paper bag and were stored in a cool and dry environment at room conditions, out of direct sunlight and under consistent temperature. Once all seeds were collected, the plant was harvested. Plants that did not produce seeds were collected at the end of their annual cycle as well. These data allowed us to determine the reproductive individual rate, and separate the individuals that produced at least one seed from those that did not produce any seed. Per capita plot-population growth rate was calculated following the Ricker’s (*r*) equation: *r* = ln(N_t_/N_t−1_) (for an example, see Estay et al., 2011). To avoid competition among plants, only one seedling per position was allowed to grow, and thus N_t−1_ corresponds to the number of positions per plot (Fig. S1), in which seeds were sown at the beginning of a year and N_t_ to the seed produced per plot at the end of the year (Estay et al., 2011). Thus, each plot of each year was considered as a population and the calculated per capita population growth thus reflects an unbiased measure of per capita population growth.

### Statistical analyses

For each species separately, we statistically tested two questions: (1) whether differences in the early and/or late precipitation predictability affect the different vital rate traits of the ancestral generation planted in four different years; and (2) whether vital rates of descendant generations differed from those of the ancestral generation, and if these differences were induced by the predictability treatment.

For the first question, we tested how predictability treatments affect the seedling emergence, the plant survival during the early and the late stage, and the reproductive individual rate, all as binary dependent variables. To this aim, Generalized Linear Mixed-effect Models (GLMMs) using a binomial distribution were conducted for each species separately, using the *lme4* package (Bates et al., 2015) in R 3.3.1 version (R Development Core Team, 2016). To test the effects on the seedling emergence and plant survival during the early stage (i.e., early survival), we included early treatment (less predictable vs more predictable), year (2012, 2013, 2014, 2015) and their two-way interaction as fixed factors, and plot as a random factor. The interaction between year and treatment led us to determine whether the treatment effect was consistent or change among years. Because survival during the late stage (late survival) and reproductive individual rate could be affected by both early and late treatment, we therefore for these metrics used the model as previously described, but we also included late treatment (less predictable vs more predictable) as an additional fixed factor, as well as its two- and three-way interactions with the other factors. To test the effects of precipitation predictability on the per capita population growth rate, as a continuous dependent variable, LMM were conducted, including early treatment, late treatment, year and their two- and three-way interactions as fixed factors and plot as random factor. As a response variable with Gaussian distribution, the population growth rate variable for normality and homogeneity of variance using Shapiro–Wilks and Bartlett tests were tested. To meet the normality of residuals assumption, response variables were transformed (see transformations in Table 1). In the presence of heteroscedasticity, and if a transformation did not result in homocedasticity, weighted least square regressions were applied.

**Table 1:** Results of the GLMM and LMM models showing the effects of the predictability treatment on vital rates variables and on the population growth rate.

Treatment effects on ancestors (G_0_)									
Response variable	Parameter	Chi-sq	Df	*P-value*		Estimates ± SE/Figure	Marginal *R*^2^	Conditional *R*^2^	*N*
*Papaver rhoeas*									
Seedling emergence	Early	2.53	1	0.112			19.84	22.49	3472
	Year	351.39	3	<0.001	***				
	Early × Year	20.50	3	<0.001	***	Fig. 2A			
Early plant survival	Early	3.06	1	0.080	·		32.68	35.88	1304
	Year	211.88	3	<0.001	***				
	Early × Year	26.67	3	<0.001	***	Fig. 2B			
Late plant survival	Early	15.67	1	<0.001	***		55.17	57.75	831
	Late	1.77	1	0.183					
	Year	169.62	3	<0.001	***				
	Early × Late	2.91	1	0.088	·				
	Early × Year	22.87	3	<0.001	***	Fig. 2C			
	Late × Year	10.14	3	0.017	*				
Population growth rate^#^	Early [M]	7.49	1	0.006	**	−6124.0 ± 2238.3	25.48	40.72	64
*Onobrychis viciifolia*									
Seedling emergence	Early	0.100	1	0.752			1.60	3.23	2576
	Year	22.16	3	<0.001	***				
	Early × Year	8.23	3	0.042	*	Fig. 2D			
Early plant survival	Early [M]	4.36	1	0.037	*	−0.350 ± 0.168	5.19	8.18	1362
Reproductive individual rate	Early [M]	8.25	1	0.004	**	−0.692 ± 0.241	7.99	17.40	590
Population growth rate^*f*^	Early [M]	8.33	1	0.004	**	−121618 ± 42130	30.69	48.15	64
	Late [M]	6.56	1	0.010	*	−107939 ± 42130			

**Notes:**

Results of the GLMM and LMM models showing the effects of the predictability treatment on vital rates variables and on the population growth rate of the ancestral generation of *Papaver rhoeas* and *Onobrychis viciifolia*. Treatment effects (‘Early’ and ‘Late’ refer to early and late treatment), year and their interactions of the reduced models are shown. Estimates ± SE are given for significant main factors and square brackets indicate the treatment level (M: more predictable treatment) to which the estimate corresponds (e.g., negative estimates indicate that M is significantly lower than L), and the figure number is given for each significant interactions. Marginal and conditional *R*^2^ (in %) are reported for all reduced models. Sample size (*N*) of each model is included. For each species, transformation in the population growth rate is given below the table and must be taken into account to understand the estimates ± SE of this variable. Significant results are further indicated with asterisk (* 0.05 > *P* > 0.01; ** 0.01 > *P* > 0.001; *** *P* < 0.001).

Transformations: #^3.7; *f*^5.

To test whether descendants exhibited a transgenerational response with respect to the imposed treatments, data collected during 2013, 2014, and 2015 was used, when plants of the descendant generations (G_1_, G_2_, and G_3_) were grown in the same plots as plants of the ancestral generation (G_0_). Thus, in ancestors, year corresponds to the year of sowing, and in descendants it also refers to the *N*th descendant generation: 2013 is equivalent with G_1_, 2014 with G_2_, and 2015 with G_3_. Since in 2012 no descendants existed, plants growing in 2012 were not included in these analyses. Growing descendant and ancestral generations together in the same plot, thus allows to detect potential transgenerational responses done by descendants even in the presence of potential huge differences among years and plots, since differences between ancestors and descendants can be analyzed on the plot level. Here, the same variables as in the first question were analyzed using GLM, GLMM and LMM, and included generation (ancestral vs descendant), early treatment, late treatment, year and their two-, three-, and four-way interactions as fixed factors, and plot and the ID of the maternal line as random factors.

In all statistical analyses, the most parsimonious model was determined using stepwise backward elimination. Marginal and conditional *R*^2^ values were calculated for all reduced models using the *rsquared* function in the *piecewiseSEM* package (Lefcheck, 2015). Post-hoc tests (*lsmeans* package; Lenth, 2016) were applied using Tukey’s HSD test, whenever there were significant main effects or interactions with factors containing more than two levels.

## Results

### Immediate effects of precipitation predictability

**Figure 2:**
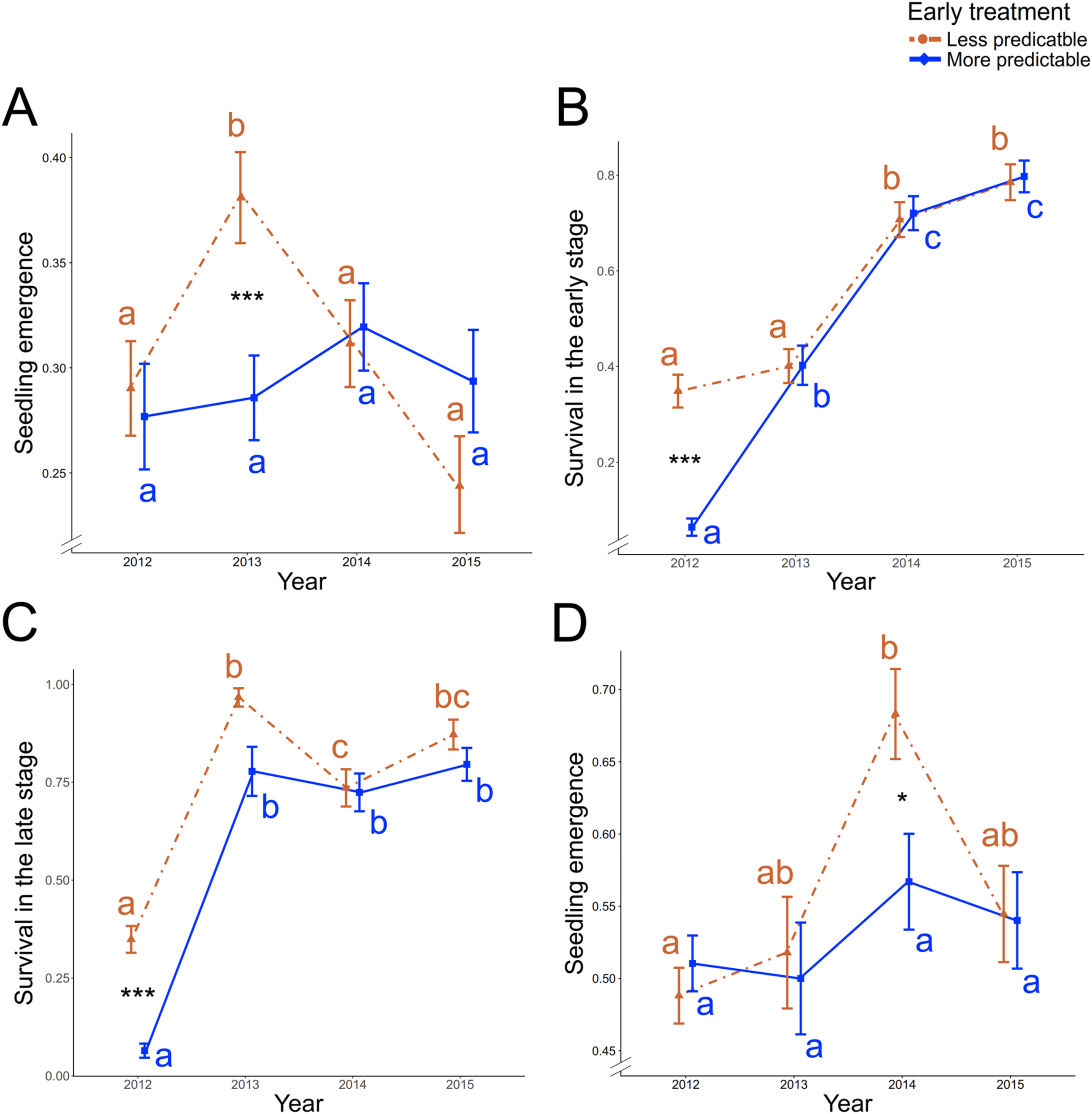
Precipitation predictability-treatment and year effect on vital rate traits of ancestral generation. Significant early treatment × year two-way interaction (A) on the seedling emergence, (B) on the survival during the early stage, and (C) on the survival during the late stage in ancestors of *P. rhoeas*. (D) Significant two-way interaction effect between early treatment and year on the ancestors’ seedling emergence in *O. viciifolia*. Other significant early and/or late predictability-treatment (without interaction with year parameter) effects are shown in the Table 1. Red and dashed lines represent the less predictable treatment and blue and solid line represent the more predictable treatment. Means ± SE is shown for each early treatment × year combination. Significant post-hoc contrasts between less and more early predictable treatment within each year are indicated with asterisk (*0.05 > *P* ≥ 0.01; ***P < 0.001). Colored letters represent post-hoc contrast differences across years in each treatment level (red: less predictable treatment; blue: more predictable treatment).

In the ancestral generation of *P. rhoeas*, which were grown all four years, an average of 30.24 ± 0.78 SE % of the seeds emerged across all years and treatments. There was a significant early predictability treatment × year two-way interaction effect on the seedling emergence (Table 1). The seedling emergence was significantly higher in 2013 in the less predictable treatment (post-hoc contrast: *P* < 0.001; Fig. 2A), and no significant treatment differences existed in the other years (*P* ≥ 0.96 in all three post-hoc contrast; Fig. 2A). Moreover, in the less predictable treatment, seedling emergence was higher in 2013 than in 2012, 2014 and 2015 (*P* ≤ 0.009 in all three post-hoc contrast), but no significant differences existed among other years or with the more predictable treatment (*P* ≥ 0.19 in all post-hoc contrasts). On average, 50.23 ± 0.01 SE % of the emerged seeds survived during the early stage. For survival during the early stage, there was a significant early treatment × year two-way interaction effect (Table 1). In 2012, the survival during the early stage was significantly higher in the less predictable treatment (post-hoc contrast: *P* < 0.001; Fig. 2B), and no significant treatment differences existed in the other years (*P* ≥ 0.79 in all post-hoc contrasts; Fig. 2B). Moreover, in the less predictable early treatment, survival during the early stage was lower in 2012 and 2013 than in 2014 and 2015 (*P* ≤ 0.01 in all four post-hoc contrasts). In the more predictable early treatment, the lowest survival during the early stage was found in 2012 (*P* ≤ 0.01 in all three post-hoc contrasts), and survival in 2013 was lower than in 2014 and 2015 (*P* ≤ 0.01 in all two post-hoc contrasts).

Of the plants surviving the early stage across all years and treatments, 53.19 ± 0.02 SE % were also survival during the late stage. There were two significant two-way interactions: early treatment × year and late treatment × year on the survival during the late stage effects (Table 1). The survival in the late stage was higher in the less predictable early treatment of 2012 (post-hoc contrast: *P* < 0.001; Fig. 2C), while no significant differences existed between early predictability treatments in the subsequent years (*P* ≥ 0.39 in all three post-hoc contrasts; Fig. 2C). Moreover, in both early treatment levels, survival was lower in 2012 than in other years (*P* < 0.001 in all post-hoc contrasts) and besides, survival was higher in 2013 than in 2014 in the less predictable treatment (post-hoc contrast: *P* = 0.04). There were not significant late treatment differences in the survival during the late stage in any year (*P* ≥ 0.19 in all four post-hoc contrasts; Fig. S3), but differences between years in both late treatments were found, since the lowest survival was found in 2012 (*P* < 0.001 in all post-hoc contrasts), and it was higher in 2015 than in 2014 in the less predictable treatment (post-hoc contrast: *P* = 0.01). Moreover, a marginally significant effect was found in the early × late two-way interaction (χ_1_^2^ = 2.91, *P* = 0.088), representing a significant higher proportion of survival during the late stage in LL and LM than in ML (two post-hoc contrasts: *P* < 0.001).

From the individuals of *P. rhoeas* that finally survived across all years and treatments, 86.68 ± 0.02 SE % were reproductive individuals (i.e., producing at least one seed). The highest reproductive individual rate was found in LL (88.00 ± 0.03 SE %) and the lowest in LM (85.91 ± 0.03 SE %), but no significant differences in treatment or treatment interactions were found (χ_1_^2^ ≤ 3.48, *P* ≥ 0.32). Finally, the per capita plot-population growth rate in *P. rhoeas* was 49% significantly higher in the less predictable early treatment (Table 1) and no significant differences were found in the late treatment (χ_1_^2^ = 0.90, *P* = 0.342).

In the ancestral generation of *O. viciifolia*, which were grown all four years, 53.03 ± 0.01 SE % of the seeds emerged across all years and treatments. There was a significant treatment × year two-way interaction effect on the seedling emergence (Table 1). In 2014, the seedling emerged was significantly higher in the less predictable than in the more predictable treatment (post-hoc contrast: *P* = 0.029; Fig. 2D), but no significant treatment differences existed in the other years (*P* ≥ 0.69 in all post-hoc contrasts). Moreover, in the less predictable treatment, seedling emergence was lower in 2012 than in 2014 (post-hoc contrast: *P* < 0.001), but no significant differences existed between other years or in the more predictable treatment (*P* ≥ 0.09 in all post-hoc contrasts). On average, 72.54 ± 0.01 SE % of the emerged seeds survived during their early stage. The survival during early stages was significantly higher in the less predictable treatment (Table 1).

During the late stage, 99.32 ± 0.00 SE % of the plants survived until harvesting, and factor levels exhibited no variance. Across all years and treatments, 35.76 ± 0.02 SE % of individuals survived produced at least one seed. The reproductive individual rate was affected by the early treatment (Table 1), being 41% significantly higher in the less predictable (44.63 ± 0.03 SE %) than in the more predictable (26.15 ± 0.03 SE %) early treatment. However, the reproductive individual rate was not affected by the late treatment (χ_1_^2^ = 2.59, *P* = 0.108). The per capita plot-population growth rate in *O. viciifolia* was 77% and 72% significantly higher under less predictable conditions of the early (Table 1) and late treatments (Table 1), respectively.

### Differential effect of precipitation predictability between ancestors and descendants

**Figure 3:**
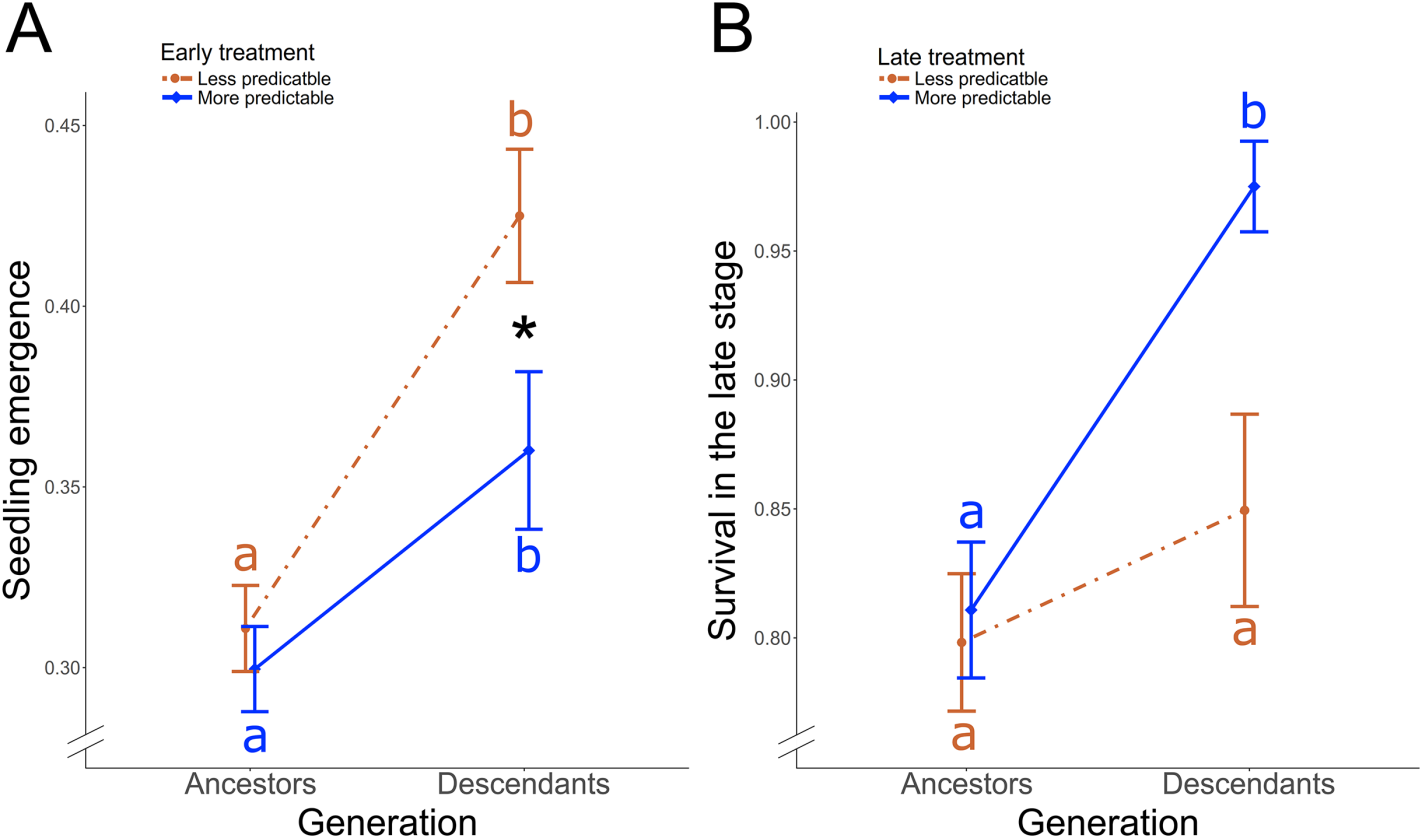
Treatment-induced transgenerational responses. In *P. rhoeas*, differences between ancestral generation and descendant generation according to (A) the early treatment on the seedling emergence, and according to (B) the late treatment on the survival during the late stage. Significant differences between more and less predictable treatment in descendants are indicated with an asterisk (*0.05 > *P* ≥ 0.01). Significant post-hoc contrasts between descendants and ancestors within each treatment are indicated with different colored letters (red: less predictable treatment; blue: more predictable treatment).

In *P. rhoeas*, the proportion of descendants’ seeds that emerged was 39.88 ± 0.01 SE %, while the proportion in ancestors was 30.52 ± 0.01 SE %. There was a significant early treatment × generation (i.e., ancestors vs descendants) two-way interaction effect on the seedling emergence (χ_1_^2^ = 4.04, *P* = 0.044; Fig. 3A). The proportion of emergence significantly increased in descendants with respect to ancestors from 31% to 42% in the less predictable treatment (post-hoc contrast: *P* < 0.001) and from 30% to 36% in the more predictable treatment (post-hoc contrast: *P* < 0.001). Thus, while no treatment differences were found in ancestors, a significantly higher proportion of descendant seedlings emerged under less predictable than under more predictable conditions (post-hoc contrast: *P* = 0.049; Fig. 3A). Early treatment did not yield significant differences between ancestor and descendant survival in the early stage (χ_1_^2^ = 1.50, *P* = 0.22). However, there was a late treatment × generation two-way interaction effect on the survival in the late stage (χ_2_^2^ = 4.10, *P* = 0.043; Fig. 3B). Descendants in the more predictable treatment exhibited significantly higher survival during the late stage (97.5%) than ancestors (81%; post-hoc contrast: *P* = 0.04, Fig. 3B), while no significant transgenerational differences existed in the less predictable treatment (85% in descendants, 80% in ancestors; post-hoc contrast: *P* = 0.66). As a result, descendants survival during the late stage tended to be higher in the more predictable than in the less predictable treatment (post-hoc contrast: *P* = 0.06). Moreover, treatment did not induce significant differences between ancestors and descendants on the reproductive individual rate (χ_1_^2^ ≤ 2.20, *P* ≥ 0.14), that overall was 86.73 ± 0.01 SE %, or plot-population growth rate (χ_1_^2^ ≤ 2.01, *P* ≥ 0.37).

In *O. viciifolia*, the proportion of descendants’ seeds that emerged was 61.32 ± 0.03 SE % in the less predictable and 46.13 ± 0.03 SE % in the more predictable treatment, while the proportion of ancestors was 58.77 ± 0.02 SE % in the less predictable and 52.73 ± 0.02 SE % in the more predictable treatment. However, treatment did not induced significantly differences between ancestors and descendants (χ_1_^2^ = 1.13, *P* = 0.29). The survival in the early stage between descendants (81.12 ± 0.02 SE %) and ancestors (79.02 ± 0.02 SE %) was also not affected by the treatment (χ_1_^2^ = 0.03, *P* = 0.86). 98.36 ± 0.01 SE % of the plants surviving the early stage period also survived until harvesting, thus, survival during the late stage exhibited not enough variance to run statistical tests. The reproductive individual rate was significantly higher in descendants (68.67 ± 0.05 SE %) than in ancestors (43.01 ± 0.03 SE %; χ_1_^2^ = 3.90, *P* = 0.048), but treatment-induced differences were not found (χ_1_^2^ ≤ 0.27, *P* ≥ 0.60). Population growth rate was 16% higher in descendants but treatments did not induce significant differences between ancestors and descendants (χ_1_^2^ ≤ 0.41, *P* ≥ 0.51). However, in both, ancestors and descendants, the reproductive individual rate was lower in MM (ancestors = 24.56 ± 0.06 SE %; descendants = 45.00 ± 0.11 SE %) than in the other treatment combinations (overall in LL, LM, and ML, the reproductive individual rate was ≥36.11% in ancestors and ≥70.19% in descendants). The plot-population growth rates were negative and lower in MM (*r*_ancestors_ = −0.41 ± 1.50 SE; *r*_descendants_ = −2.26 ± 1.90 SE) compared to other treatment combinations, where they were positive (LL, LM, and ML, *r*_ancestors_ ≥ 1.76 and *r*_descendants_ ≥ 2.80).

## Discussion

In an era of rapid and less predictable weather changes (Bradshaw, 2006), models aim to project how will the future of the individuals be considering the accelerated rates of these changes (Botero et al., 2015). However, previous experimental tests of the effects of increasing weather variability and associated reduced precipitation predictability on seedling emergence and plant survival have mainly concentrated on extreme events or increased drought (Knapp et al., 2002; Rivaes et al., 2013; Bailey et al., 2017; Pardo et al., 2017). Experimentally simulating more subtle climate events, we reveal that lower precipitation predictability can actually stimulate the viability of *P. rhoeas* and *O. viciifolia*, rather than leading to increased mortality. In our experiments, plant vital rates were not negatively affected by lower precipitation predictability, in agreement with bet-hedging models (Clauss & Venable, 2000). In fact, contrary to other suggestions (Anderson, 2016), greater short-term survival and reproduction success were found under less predictable conditions, increasing the plot-population growth rate (hereafter referred as population growth). This was consistent across life stages. Moreover, transgenerational responses did not show negative effects of either lower intra- or inter-seasonal predictability. Descendants exhibited stable or greater transgenerational responses when they were subjected to less predictable conditions. The results of this experiment suggest that increased environmental unpredictability may not always negatively affect all plants.

### Effects of precipitation predictability on vital rates traits

The first phenotypic expression of plants when testing the effects of climate changes appear through seedling emergence (Donohue et al., 2010). In our experiment, seedling emergence was higher with lower precipitation predictability in 2013 for *P. rhoeas* and in 2014 for *O. viciifolia* (Figs. 2A, and 2D), but differences between treatments did not appear in other years. Although the quantity of precipitation was identical in both treatments, the less predictable treatment led to more changes on soil moisture, varying between dry and wet soils during the emergency period, due to its associated inconsistency (by experimental design). While soil warming and water scarcity generally reduces seedling emergence (Classen et al., 2010; Hoyle et al., 2013), inconsistent soil moisture (e.g., seeds experiencing a series of hydration and dehydration) could promote a greater seed activation or dormancy break (Baskin & Baskin, 1982; Fenner & Thompson, 2005; Walck et al., 2011). Therefore, seedling emergence could be favored if precipitation events are more variable and/or less predictable (Clauss & Venable, 2000) as this is among the most effective seed dormancy-breaking factors (Walck et al., 2011). As differences were not found in every year, the effects of predictability on seedling emergence were not very consistent but suggest that seed germination may be sensitive and may benefit from lower environmental predictability (Fay & Schultz, 2009). Furthermore, seeds also emerged faster under less predictable precipitation (March-Salas et al., 2018, unpublished data). Greater and earlier seedling emergence could provide competitive and fitness advantages (Cohen, 1967; Gremer, Kimball & Venable, 2016; March-Salas et al., 2018, unpublished data), such as maximizing the available resources or allowing plants to grow larger before reproduction (Donohue et al., 2010).

In previous literature some authors found that precipitation variability and the absence of static water availability can promote seed activation (Gremer & Venable, 2014). They also found that post-germination growth and survival were reduced in variable and less predictable environments. In contrast, we found that lower precipitation predictability enhanced the overall survival during the early stage in *O. viciifolia* (Table 1) and the survival during the early and the late stage in *P. rhoeas* in 2012 (Figs. 2B, and 2C). The effect on *O. viciifolia* was consistent and independent of the year. However, the effect of year in *P. rhoeas* shows fewer consistency and it could be explained by the lower proportion of survived plants in 2012 compared to 2013–2015. Survival in the early stage was at least 19% significantly lower in 2012 than in other years, and survival in the late stage was at least 52% significantly lower in 2012 than in other years. This is probably explained by the drier late spring and summer in 2012 compared to other years (Fig. S4). This suggests that differences between predictability levels could increase when mean precipitation is lower or when survival is lower. Other hypotheses can arise from the life strategy of *P. rhoeas*. This annual species has to produce seeds in a single year, and, consequently, their populations could be more plastic since they should survive under all environments, including adverse conditions (Friedman & Rubin, 2015).

Survivorship can increase at the expense of seed production, but plants that arrive to the end of their life-cycle aim to invest their resources in offspring production (Morris et al., 2008). In *P. rhoeas*, no differences were found in the reproductive individual rates. This can be explained because, in all treatments, more than 86% of surviving plants produced seeds, since annual species should invest all their reproductive efforts in their sole year of life (Primack, 1979). However, as perennial species, *O. viciifolia* can choose between an immediate performance, or delay seed production, keeping resources for the following year. We found 41% higher reproductive individual rate in the less predictable than in the more predictable treatment. This result suggests that, in the short-term, *O. viciifolia* may use bet-hedging strategies to minimize risks of future reduced predictability (Childs, Metcalf & Rees, 2010), investing more resources in seed production during their first year. Therefore, subtle precipitation changes generate uncertainties that could be solved by promoting rapid reproductive response in order to ensure plant viability, as a conservative strategy.

### Plant viability under subtle vs extreme precipitation changes

It is widely assumed that climate change is already modifying the distribution of organisms and endangering the future of many species (Thomas et al., 2004), but studies testing whether and how lower environmental predictability affects plant vitality are scarce (van de Pol et al., 2010). In *P. rhoeas*, the population growth rate increased significantly in the less predictable (mean ± standard error: 3.31 ± 0.56 SE) vs the more predictable (1.69 ± 0.74 SE) early treatment. *Onobrychis viciifolia* also had higher population growth rates in the less predictable (L) compared to the more predictable (M) treatment, but during both early (L = 2.62 ± 0.69 SE; M = 0.61 ± 0.85 SE) and late stages (L = 2.52 ± 0.69 SE; M = 0.71 ± 0.85 SE). This is an experimental evidence that lower environmental predictability does not necessarily reduce population growth rates, but can actually increase them, as recently suggested (Lawson et al., 2015). Thus, not all environmental changes will depress viability and reproductive components, as suggested by Anderson (2016). However, our experiment simulates precipitation predictability that does not exceed the current range of precipitation variance, and omits the increasing existence of extreme events (Katz & Brown, 1992; Easterling et al., 2000). Therefore, short-term population growth rates could be favored if current environmental changes are stabilized, since species may experience an increase of plasticity if new environmental conditions fall within the plastic tolerance limits of several species (Ghalambor et al., 2007).

These results also suggest that at least some plants will be able to cope with gradual climatic trends and subtle precipitation fluctuations. Environmental variability was identified as one of the major drivers of current global change more than 20 years ago (Karl, Knight & Plummer, 1995), promoting frequent unpredictable weather events. Thus, another hypothesis to consider might be that since plants have already been growing under increasing variability and reduced predictability during recent decades, they consequently may already have become preadapted to higher than to lower environmental variability and unpredictability, allowing their offspring to persist to increasing variability and unpredictability even future extreme events (Chevin & Hoffmann, 2017). Therefore, in the absence of non-catastrophic events, subtle less predictable conditions could promote rapid adaptation or increasing plasticity, and they may increase vitality and population growth rates.

### The relevance of precipitation predictability on plants’ early life

The effects of precipitation predictability were higher during the early stage than during the late stage (Table 1), as suggested by other studies (Burton & Metcalfe, 2014). Seedling emergence and survival in early stages could only be affected by early treatment and not by late treatment (see Fig. 1). However, the effects of early treatment on these traits were remarkable for both species (Table 1). Furthermore, in *P. rhoeas*, survival during the late stage was affected by the interaction of year with early treatment and with late treatment, but while early treatment induced differences in some years (Fig. 2C), there were not any significant differences between late treatment levels (Fig. S3). The reproductive individual rate in *O. viciifolia* was significantly affected by the early treatment but not by the late treatment, showing that lower early precipitation predictability entail more reproductive individuals. These results show that environmental conditions in early stages strongly influence later performance (Jonsson & Jonsson, 2014) in terms of survival and reproductive efforts.

The consistent effects of environmental predictability on early life could potentially entail cascading effects, changing the behavioral dynamics on subsequent life-cycles (Post et al., 2008). This is corroborated by the higher population growth rate on early treatment. While the effect of precipitation predictability in *P. rhoeas* only appears during the early treatment (Table 1) and not during late treatment, both early and late predictability influence the population growth of *O. viciifolia*. However, the strongest effect was found during the early treatment (Table 1). This trend suggests that predictability conditions during early development may change subsequent phenotypic expressions (Burghardt et al., 2015), preparing plants for long-term fitness consequences (Cam, Monnat & Hines, 2003), and potentially influencing the response of future generations (Burton & Metcalfe, 2014).

### Transgenerational responses

The effects of precipitation-predictability on transgenerational performance were found in *P. rhoeas* (an annual species) but not in *O. viciifolia* (a perennial species). This could indicate that short-lived species may be more sensitive to predictability than long-lived species (Morris et al., 2008) and that the life strategy of *O. viciifolia* could delay potential selective pressures. However, studies with more different annual and perennial species are needed to corroborate this hypothesis. We found that, under both more and less predictability treatments, descendants of *P. rhoeas* had significantly greater seedling emergence than ancestors (Fig. 3A). This result shows that, as seedling emergence is the earliest life stage, it is a process very susceptible to be subjected to selective pressures (Donohue et al., 2010), as other studies found under warmer parental temperatures (Bernareggi et al., 2016). The proportion of emerged seeds increased steeply and was significantly higher in descendants subjected to less predictable precipitation (Fig. 3A). The rapid transgenerational response in seedling emergence could suggest an increase of phenotypic plasticity (Reed et al., 2010; Merilä & Hendry, 2014; March-Salas et al., 2018, unpublished data) that was quicker under less predictable conditions. In variable and less predictable environments, traits may evolve to either enhance the emergence of seeds in response to cues predicting favorable conditions or limit the risk of seedling emergence under unfavorable conditions by increasing variance in the timing of emergence (Gremer, Kimball & Venable, 2016). Evidence of adaptation and selective processes on seedling emergence were previously described (Donohue et al., 2010), but this experiment shows empirical evidence of transgenerational processes that occurred due to precipitation-predictability levels.

Species survival may depend as much on keeping pace with moving climates as the climate’s ultimate persistence (Loarie et al., 2009). Over time, the persistence of higher predictability treatments significantly increase the survival on descendants of *P. rhoeas* compared to their ancestors (Fig. 3B), while under less predictable treatment, survival increased but not significantly. This agrees with a slower evolutionary response under environmental variability on long-term survival (Kingsolver & Buckley, 2015). The results in *P. rhoeas* point out to a rapid response to immediate changes in precipitation predictability (Bradshaw, 2006), since transgenerational response in terms of seedling emergence, which occurs during early life, was affected by early treatment (Fig. 3A), and transgenerational response in terms of late survival was affected by late treatment (Fig. 3B).

## Conclusions

Our experiment suggests that some plants would be able to cope with reducing environmental predictability, at least in the absence of extreme events. Still, the transgenerational responses to less predictable environments that we found here suggest that plants might be preadapted to increasing variability and unpredictability (Chevin & Hoffmann, 2017), but studies testing this hypothesis are needed. The effect of subtle reductions in precipitation predictability was mostly positive, or at least not negative, in every vital trait and along the entire plant’s life-cycle and for both species. This shows that (1) the effect was consistent, (2) the effect of environmental predictability is immediately observed, and (3) it can trigger changes in multiple life stages, leading to potential increases in population growth rates instead of the expected decline under an important component of climate change (Clark et al., 2011). Moreover, since the yield of future ecosystems could be affected by small shifts in rates of seedling emergence and establishment in certain species (Classen et al., 2010), these results may be also very relevant for ecosystem productivity. We also found that the effects were higher if predictability acts during early life, and show that plants behavior during first phases of life could lead to a benefit drag throughout life, with potential consequences in plants population dynamics. In summary, this study suggests tolerance capacities of some plants to short-term reduced predictability. If the increase in environmental variability and associated reduced predictability is not eventual and rather a progressive and gradual trend, plastic responses could be acquired with time and may reduce the impact of future catastrophic and non-catastrophic climatic scenarios, allowing plants to persist across their current plasticity range.

## Supplemental Information

10.7717/peerj.6443/supp-1 Supplemental Information 1 Supplemental Material. Supplementary text showing the species description

10.7717/peerj.6443/supp-2 Supplemental Information 2 Experimental system located at ‘El Boalar de Jaca’ (Jaca, Huesca, Spain). Two seeded plots of 1.2 × 6.0 m were established in each of 16 enclosures: one for *P. rhoeas*, and another one for *O. viciifolia*. A schema and a photograph showing the layout of the seeding plots of the experimental system. Twenty eight seeding positions (red dots) were established, each located at 40 cm from the closest seeding position and from the limits of the seeding plot. This design blocks potential competition among experimental seedlings.

10.7717/peerj.6443/supp-3 Supplemental Information 3 Plants were exposed to more or less predictable environmental conditions. Plants were exposed to more or less predictable environmental conditions (shown period: 04/06/2012 to 01/07/2012). The graph includes the total precipitation (in mm; sum of irrigation and natural precipitation). Solid line corresponds to more predictable and dashed line to the less predictable precipitation. Average precipitation (thin gray line) measured over the extent of the entire experiment (2012–2015) was identical between treatment levels (c^2^ < 0.001, *P* = 0.992), and the variance in daily precipitation was significantly higher in the less predictable treatment (χ^2^ = 605.49, *P* < 0.001).

10.7717/peerj.6443/supp-4 Supplemental Information 4 Two-way interaction effect between late treatment and year on the survival during the late stage in the ancestral generation of *P. rhoeas*. Two-way interaction effect between late treatment and year on the survival during the late stage in the ancestral generation of *P. rhoeas*. Red and dashed lines represent the less predictable treatment and blue and solid line represent the more predictable treatment. There was not significant differences at post-hoc contrasts between less predictable and more predictable early treatment within any year. Colored letters represent post-hoc contrast differences across years in each treatment level (red: less predictable treatment; blue: more predictable treatment).

10.7717/peerj.6443/supp-5 Supplemental Information 5 Monthly natural precipitation and average temperature in each year in Jaca (Huesca, Spain). Monthly natural precipitation and average temperature in each year in Jaca (Huesca, Spain), where experiment was conducted. A. Monthly precipitation (in mm) in each year. B. Average monthly temperatures (in °C) in each year. Colored lines represent different experimental years (2012–2015).

10.7717/peerj.6443/supp-6 Supplemental Information 6 Raw data. Raw data applied for data analyses and preparation for Results, Figures, and Tables.

## Additional Information and Declarations

### Data Availability

The following information was supplied regarding data availability: Raw data is available in the Supplemental Materials.
