# Supplementary material for: A multi-year experiment shows that lower precipitation predictability encourages plants’ early life stages and enhances population viability
Source: PeerJ. 2019 Mar 8;7:e6443. doi: 10.7717/peerj.6443 (PMC6410692; doi:10.7717/peerj.6443)

**SUPPLEMENTARY MATERIAL**

**Species description**

*Papaver rhoeas* is an herbaceous annual plant that grows from sea level to 1700 m a.s.l. It is widely distributed in Eurasia and northern Africa (McNaughton & Harper, 1964), where it occurs in dry to high humidity habitats (McNaughton & Harper, 1964). Its height is between 10 and 50(90) cm and its flowers are bright red and almost spherical (Franklin-Tong & Franklin, 1992). Flowering begins in May and can last until October. The fruit is a capsule that, when ripe (July–September), opens apical holes from which numerous >1 mm long, kidney-shaped seeds are dispersed (McNaughton & Harper 1964). It has a slender tap-root, sometimes with numerous secondary roots.

*Onobrychis viciifolia* is a perennial forb that grows from sea level to 2800 m a.s.l. Its native distribution covers the Mediterranean, south-eastern Europe and Siberia, but it is also widely naturalized in other parts of Eurasia, North America, Australia and New Zealand. *Onobrychis viciifolia* inhabits a broad range of climatic, soil, and humidity conditions (Carbonero et al., 2011). Its height is between 20 and 80 cm (rarely > 100 cm), and it produces dense inflorescences with 10-100 pink, zygomorphic, hermaphroditic flowers. Its flowering period is from May to August. The fruit is a small single-seeded pod. *Onobrychis viciifolia* has a deep taproot (reaching >1 m of depth), sometimes with numerous secondary roots and thin lateral roots (Mohajer et al., 2012).

**Figure S1:** Experimental system located at ‘El Boalar de Jaca’ (Jaca, Huesca, Spain). Two seeded plots of 1.2 × 6.0 m were established in each of 16 enclosures: one for *P. rhoeas*, and another one for *O. viciifolia.* A schema and a photograph showing the layout of the seeding plots of the experimental system. 28 seeding positions (red dots) were established, each located at 40 cm from the closest seeding position and from the limits of the seeding plot. This design blocks potential competition among experimental seedlings.

***
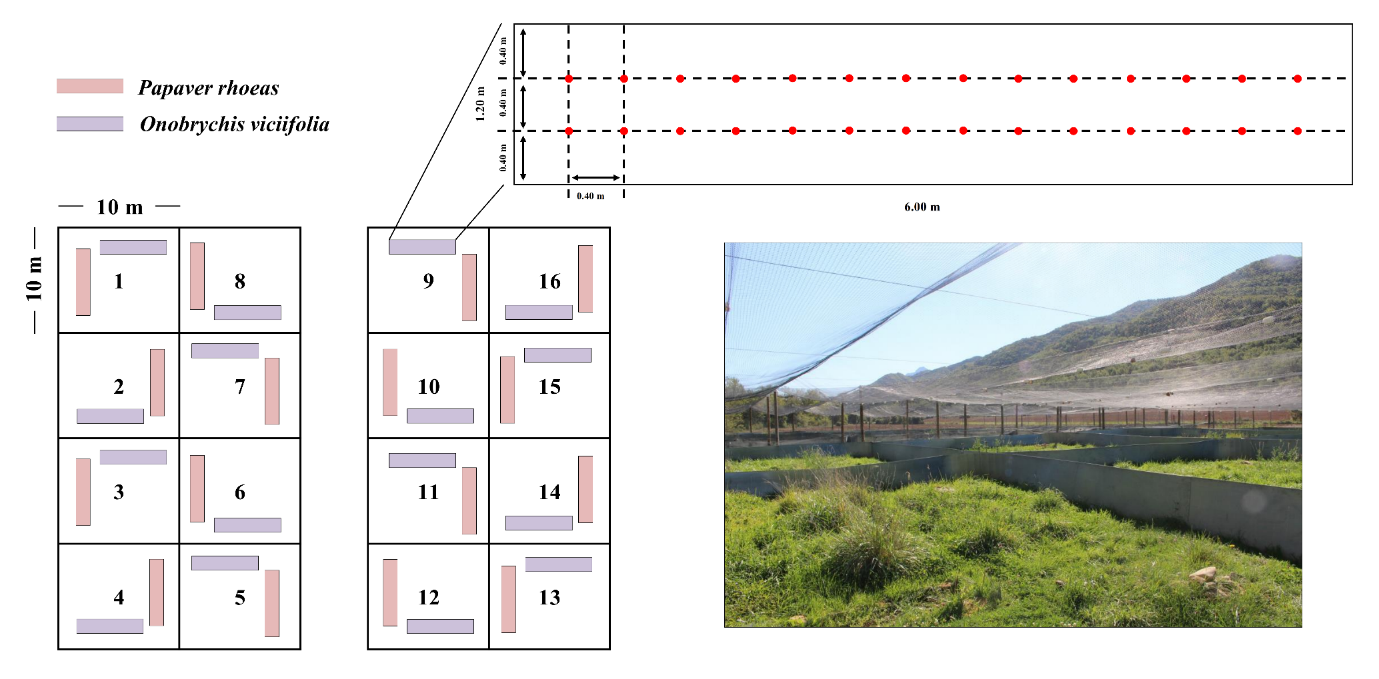
***

**Figure S2:** Plants were exposed to more or less predictable environmental conditions (shown period: 04/06/2012 to 01/07/2012). The graph includes the total precipitation (in mm; sum of irrigation and natural precipitation). Solid line corresponds to more predictable and dashed line to the less predictable precipitation. Average precipitation (thin grey line) measured over the extent of the entire experiment (2012-2015) was identical between treatment levels (χ^2^ < 0.001, *P* = 0.992), and the variance in daily precipitation was significantly higher in the less predictable treatment (χ^2^ = 605.49, *P* < 0.001).

***
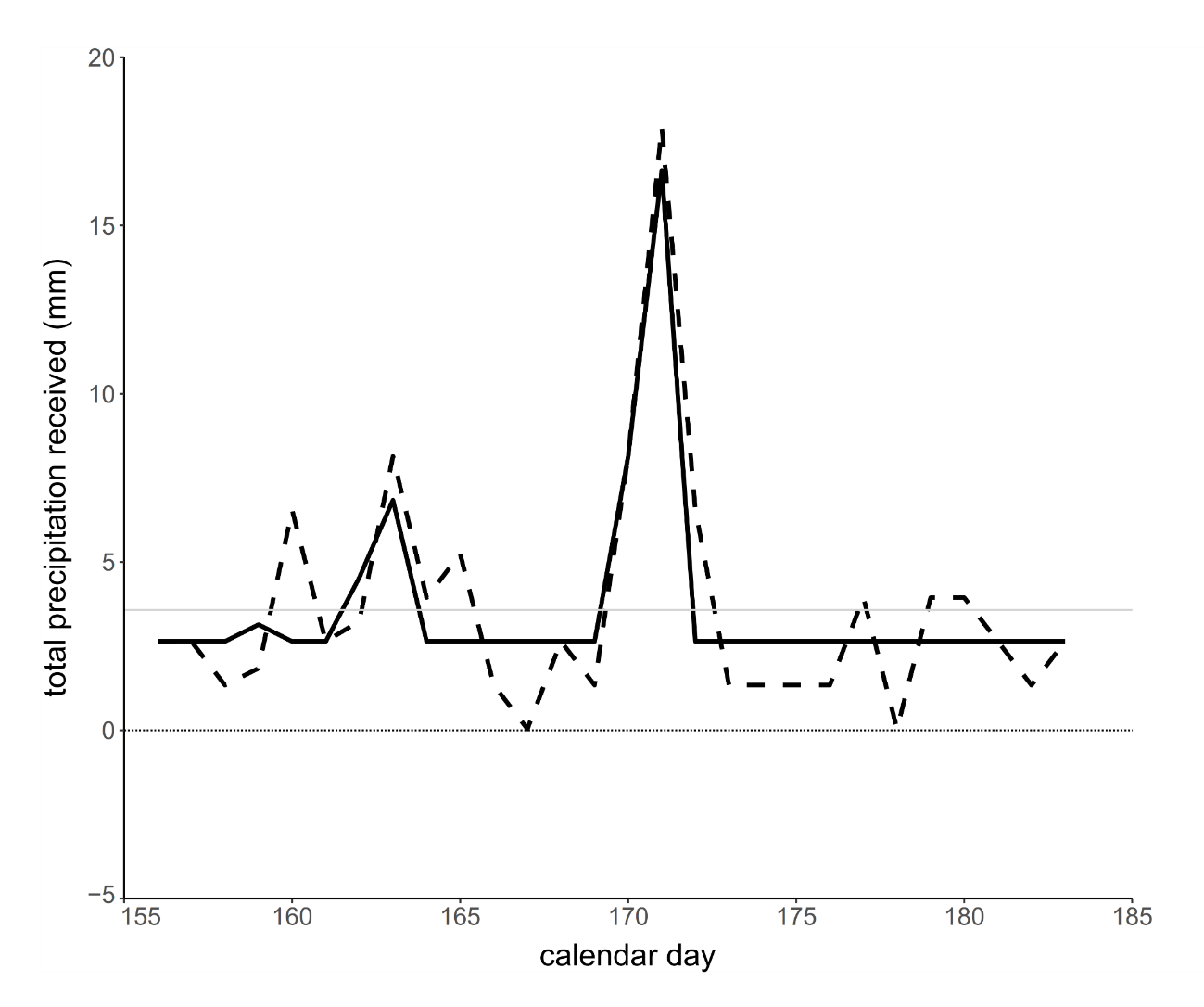
***

**Figure – S3:** Two-way interaction effect between late treatment and year on the survival during the late stage in the ancestral generation of *P. rhoeas*. Red and dashed lines represent the less predictable treatment and blue and solid line represent the more predictable treatment. There was not significant differences at post-hoc contrasts between less predictable and more predictable early treatment within any year. Colored letters represent post-hoc contrast differences across years in each treatment level (red: less predictable treatment; blue: more predictable treatment).

**
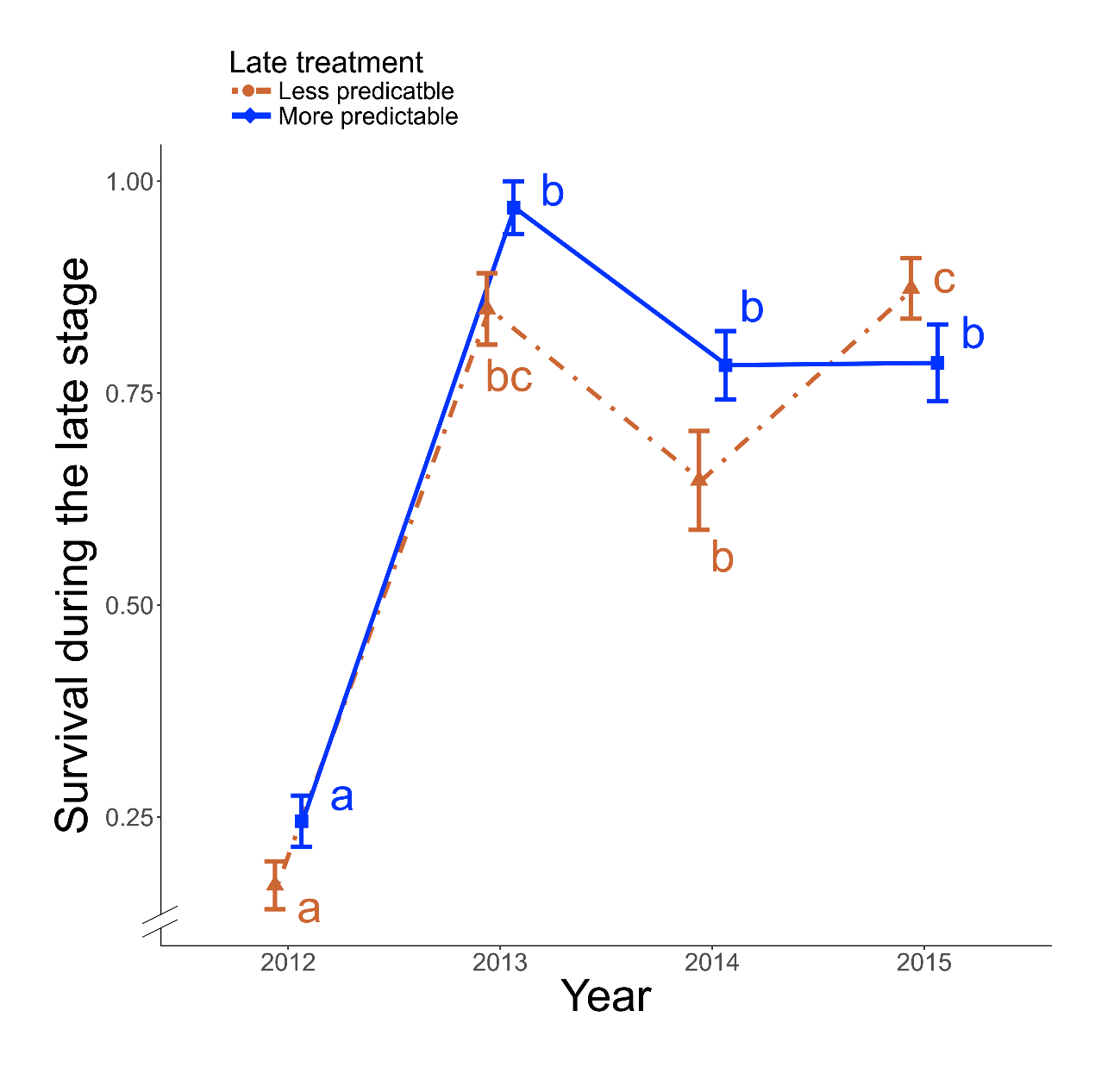
**

**Figure S4:** Monthly natural precipitation and average temperature in each year in Jaca (Huesca, Spain), where experiment was conducted. **A.** Monthly precipitation (in mm) in each year. **B.** Average monthly temperatures (in ºC) in each year. Colored lines represent different experimental years (2012-2015).


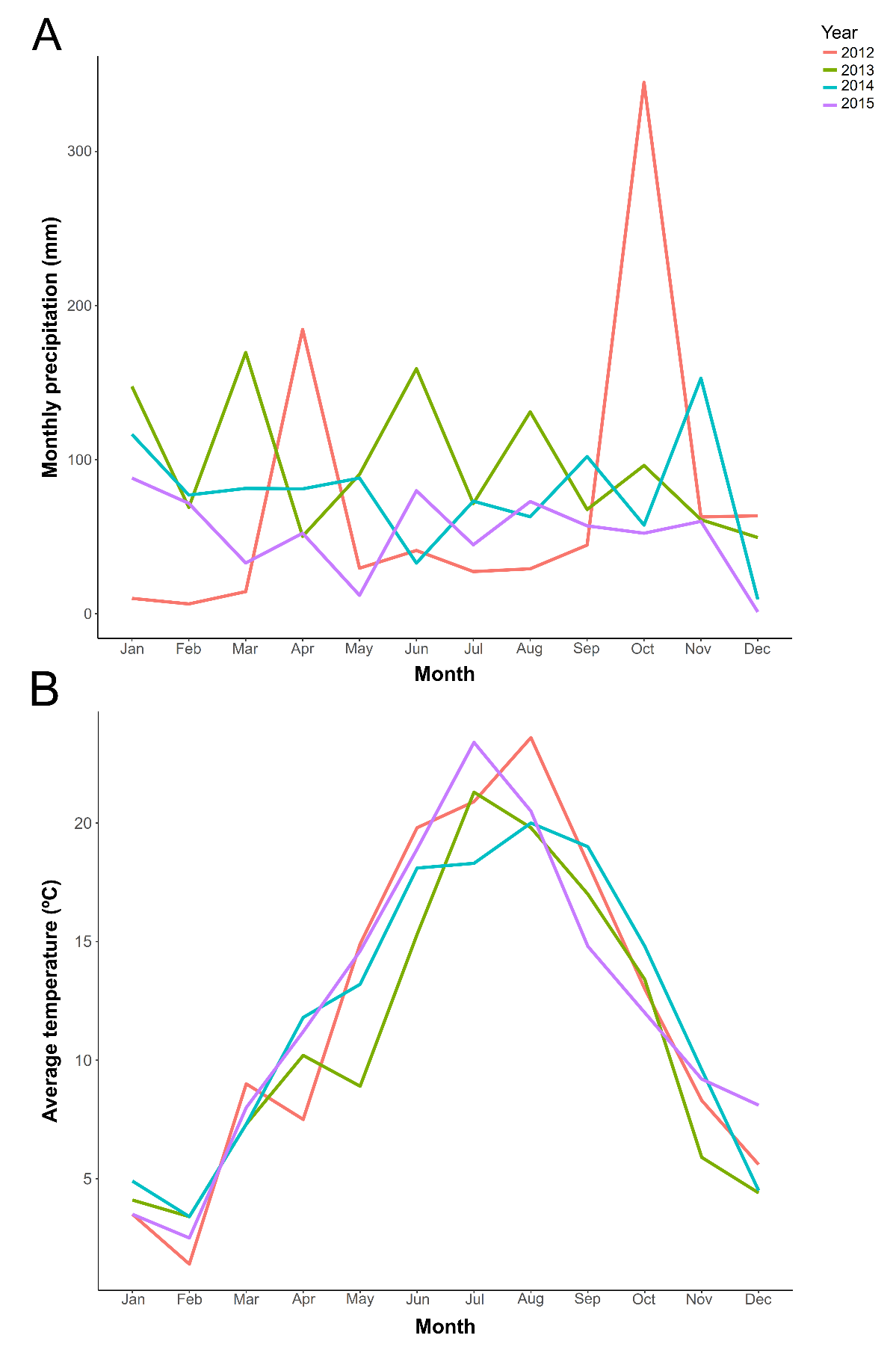

Supplement: Supplemental Information 1 — Supplementary text showing the species description [file peerj-07-6443-s001.docx]
