# Supplementary figures and images for: A multi-year experiment shows that lower precipitation predictability encourages plants’ early life stages and enhances population viability
Source: PeerJ. 2019 Mar 8;7:e6443. doi: 10.7717/peerj.6443 (PMC6410692; doi:10.7717/peerj.6443)

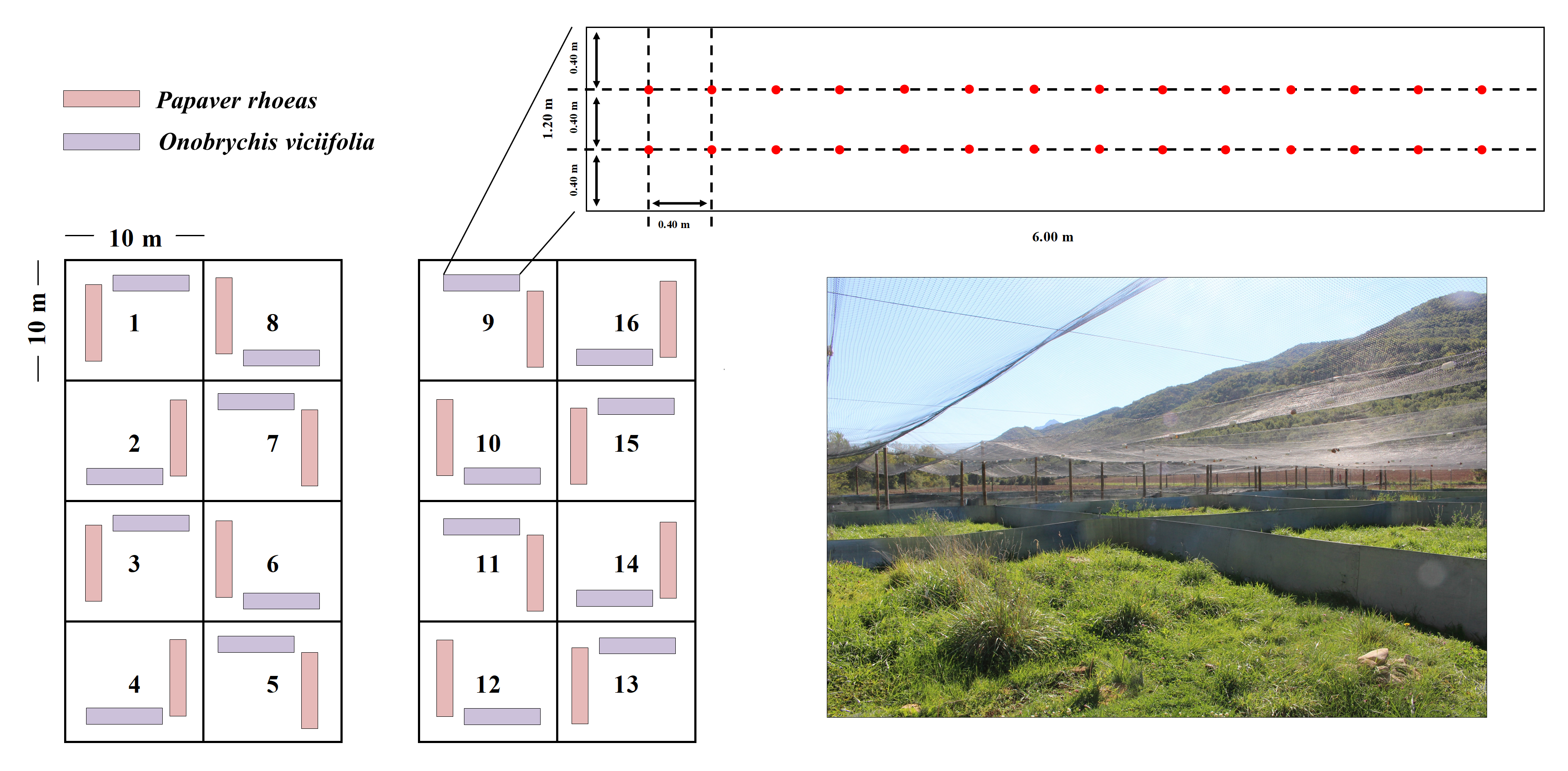

Supplement: Supplemental Information 2 — Two seeded plots of 1.2 × 6.0 m were established in each of 16 enclosures: one for P. rhoeas, and another one for O. viciifolia. A schema and a photograph showing the layout of the seeding plots of the experimental system. Twenty eight seeding positions (red dots) were established, each located at 40 cm from the closest seeding position and from the limits of the seeding plot. This design blocks potential competition among experimental seedlings. [file peerj-07-6443-s002.png]

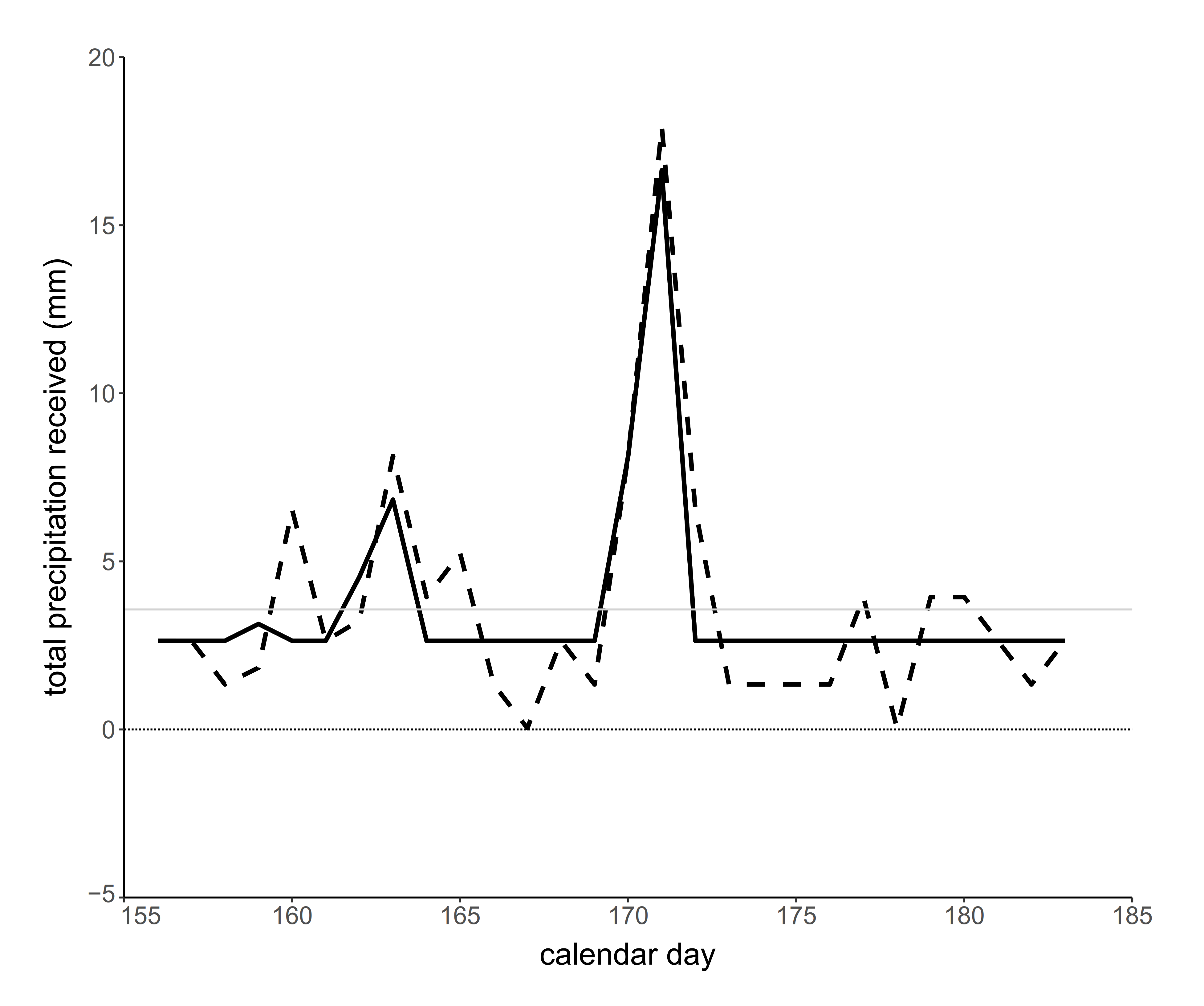

Supplement: Supplemental Information 3 — Plants were exposed to more or less predictable environmental conditions (shown period: 04/06/2012 to 01/07/2012). The graph includes the total precipitation (in mm; sum of irrigation and natural precipitation). Solid line corresponds to more predictable and dashed line to the less predictable precipitation. Average precipitation (thin gray line) measured over the extent of the entire experiment (2012–2015) was identical between treatment levels (c2 < 0.001, P = 0.992), and the variance in daily precipitation was significantly higher in the less predictable treatment (χ2 = 605.49, P < 0.001). [file peerj-07-6443-s003.png]

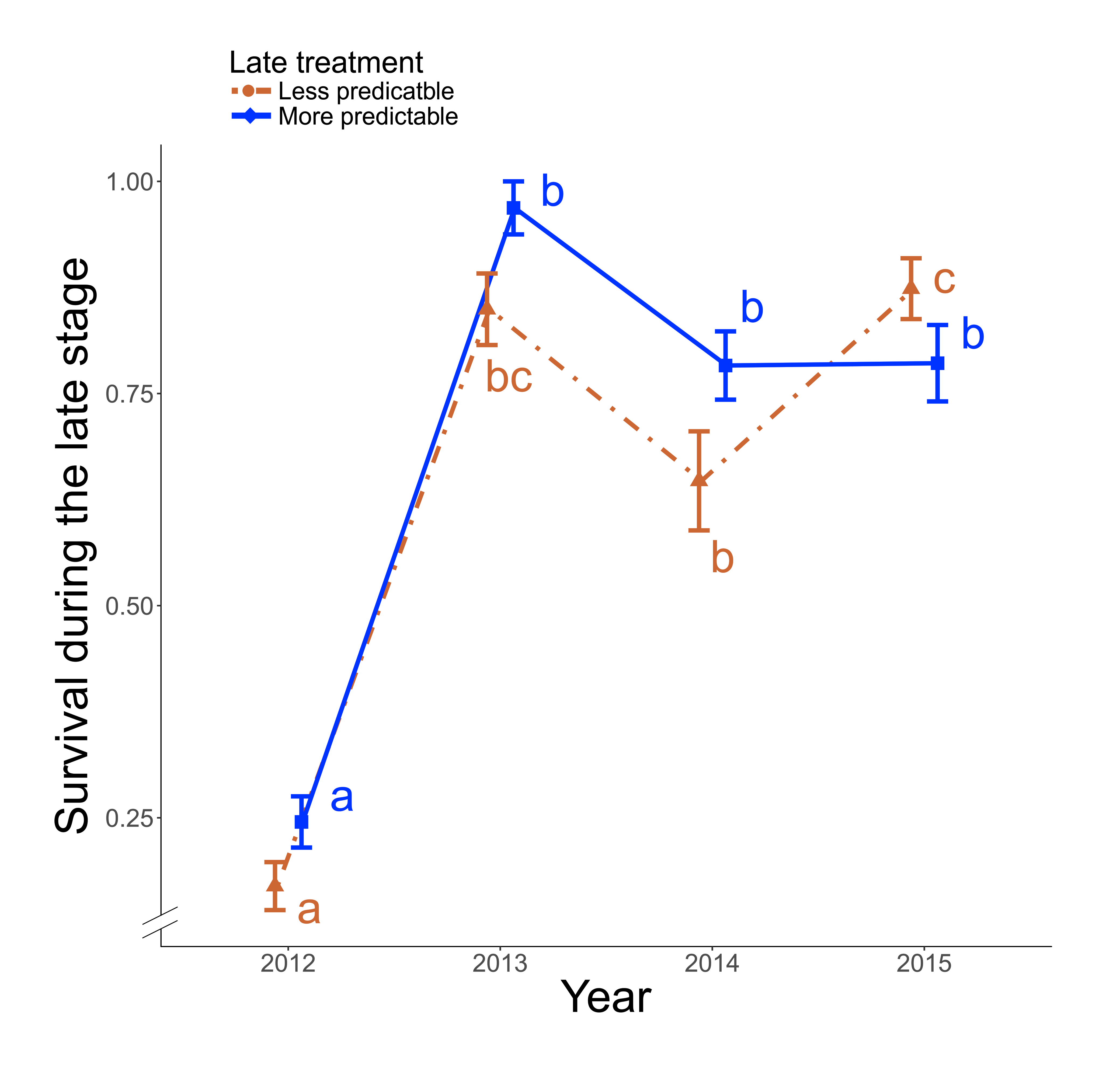

Supplement: Supplemental Information 4 — Two-way interaction effect between late treatment and year on the survival during the late stage in the ancestral generation of P. rhoeas. Red and dashed lines represent the less predictable treatment and blue and solid line represent the more predictable treatment. There was not significant differences at post-hoc contrasts between less predictable and more predictable early treatment within any year. Colored letters represent post-hoc contrast differences across years in each treatment level (red: less predictable treatment; blue: more predictable treatment). [file peerj-07-6443-s004.png]

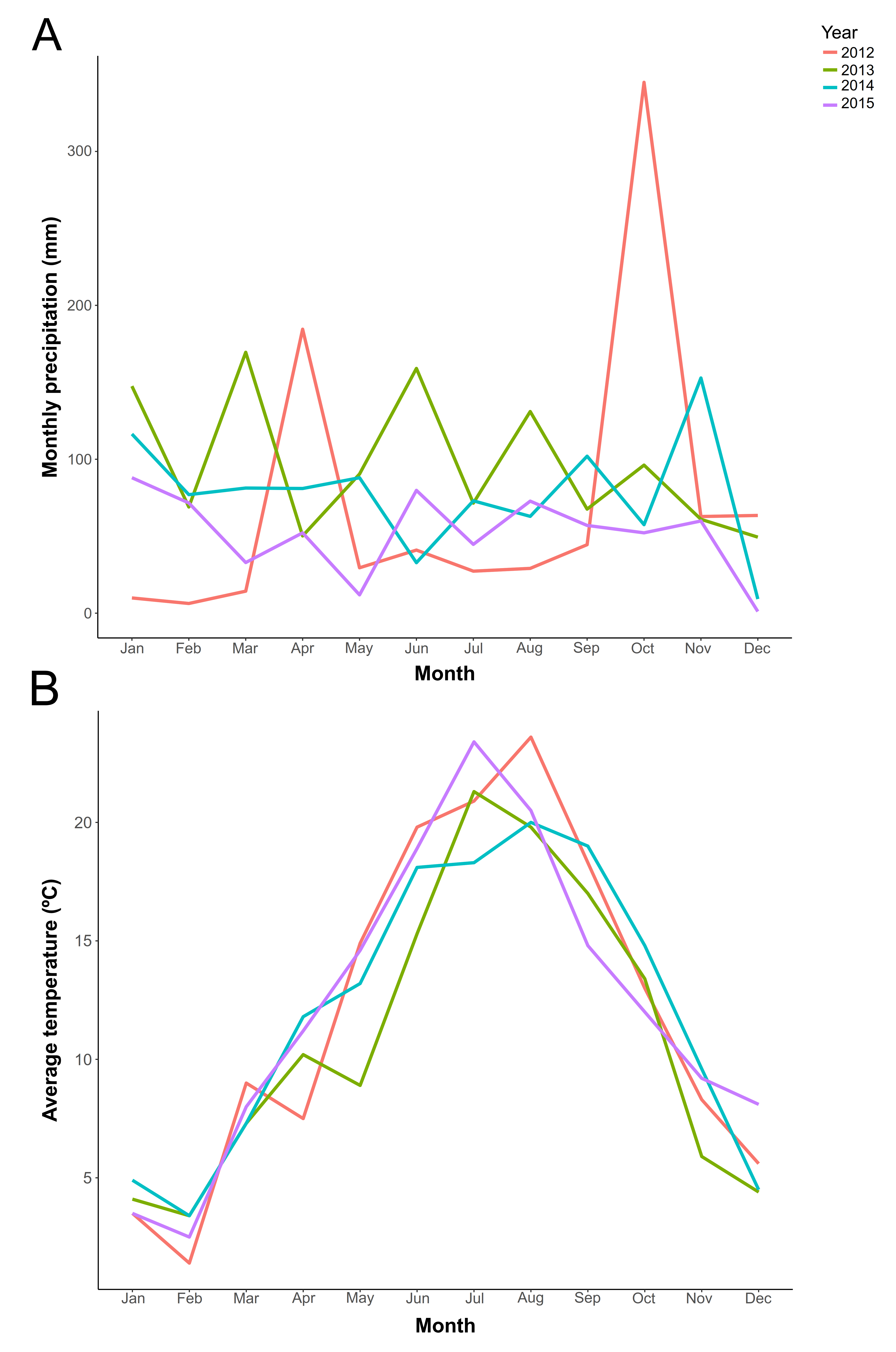

Supplement: Supplemental Information 5 — Monthly natural precipitation and average temperature in each year in Jaca (Huesca, Spain), where experiment was conducted. A. Monthly precipitation (in mm) in each year. B. Average monthly temperatures (in °C) in each year. Colored lines represent different experimental years (2012–2015). [file peerj-07-6443-s005.png]
